# Massive abdominal wall hematoma following a dog bite: a case report

**DOI:** 10.1097/RC9.0000000000000240

**Published:** 2026-03-04

**Authors:** Rema Elkalbash, Raimundas Lunevicius

**Affiliations:** a Aintree University Hospital, Liverpool University Hospitals, NHS Foundation Trust, Fazakerley, United Kingdom; b Consultant Surgeon, Mortality and Audit Governance Lead, Department of General Emergency and Trauma Surgery, Aintree University Hospital, Liverpool University Hospitals, NHS Foundation Trust, Fazakerley, United Kingdom

**Keywords:** abdominal wall hematoma, case report, computed tomography, dog bite injury, empirical antibiotics, surgical debridement, traumatic soft tissue injury

## Abstract

**Introduction and importance:**

Dog bite injuries are a major global public health concern, predominantly affecting the extremities. Abdominal wall involvement is rare and underreported. This case demonstrates a significant soft tissue hematoma following a dog bite, underscoring the importance of early imaging and surgical intervention even in the absence of coagulopathy.

**Case presentation:**

A 47-year-old obese female presented with progressive left-sided abdominal pain and bruising, 6 days post-dog bite to the left iliac fossa. Examination revealed localized ecchymosis and active bleeding, without systemic signs of infection. Contrast-enhanced CT revealed a 4.7 × 10.8 × 8.9 cm hematoma in the subcutaneous plane of the oblique abdominal muscles. The hematoma was aspirated, and necrotic tissue was debrided. The patient received tetanus prophylaxis and broad-spectrum antibiotics, with favorable postoperative recovery.

**Clinical discussion:**

Abdominal wall hematomas from dog bites are rare and may be misdiagnosed due to atypical presentation. In this case, timely CT imaging enabled prompt diagnosis and prevented complications such as compartment syndrome or abscess formation. The absence of anticoagulant use or bleeding disorders confirmed trauma as the etiology. Given the polymicrobial nature of dog saliva, empirical antibiotics were essential to prevent secondary infection.

**Conclusion:**

This case highlights the potential for severe abdominal wall injuries from dog bites. It emphasizes the significance of timely surgical intervention and the diagnostic use of CT imaging. Clinicians should include abdominal wall hematoma in the differential diagnosis for patients with localized abdominal pain and bruising after a dog bite, even without typical risk factors.

## Introduction

Animal bites pose a significant public health risk worldwide. According to the World Health Organization, dog bites account for approximately 10 million injuries annually[1]. However, this is a rough estimate, as global incidence figures have not been fully established, and many countries lack comprehensive data on dog bite injuries. There is ongoing debate regarding the true prevalence of dog attacks in England, with concerns that medical literature may overstate the actual risk^[^2,3^]^.

In the United Kingdom (UK), it is estimated that one in four individuals has been bitten by a dog at some point in their life[2]. Dog bites can lead to serious infections, including tetanus, methicillin-resistant *Staphylococcus aureus, Pasteurella*, rabies, and *Capnocytophaga*[4]. Between 1998 and 2018, there were 107 366 reported dog bite incidents in England, with approximately 27 000 individuals requiring reconstructive surgery due to dog bite-related injuries in 2018[5]. The incidence rate in the UK is estimated at 2.5 per 100 000 requiring hospital treatment and 740 per 100 000 experiencing a bite[6].

HIGHLIGHTS

- Rare case of abdominal hematoma following dog bite without coagulopathy.
- Early CT imaging led to the detection of a deep hematoma in the oblique abdominal wall.
- Surgical drainage and antibiotics led to the recovery of the patient without complications.
- Emphasize the risk of abdominal injuries when the external wound seems minor.

Several risk factors contribute to the prevalence of dog bites globally, including overpopulation of stray and domestic dogs, irresponsible pet ownership, and lack of education on human-animal interactions[7]. Beyond physical injuries, dog bite victims may suffer significant psychological trauma, and the severity and location of injuries can lead to long-term disability and disfigurement[8]. Studies suggest that 84% of severe dog bite injuries affect the upper and lower limbs, while 80.2% of all injuries involve the extremities[9]. Injuries to the thoracic, abdominal, and gluteal regions are less common[10].

We present a rare case of a massive abdominal wall hematoma resulting from a dog bite, highlighting the importance of early imaging, prompt surgical intervention, and infection prevention in managing such injuries. This case report is adhered to the SCARE checklist guidelines 2025[11].

## Case presentation

A 47-year-old female with a BMI > 35 kg/m^2^ presented with progressive left-sided abdominal pain and bruising over the left lower abdominal wall, which developed over 6 days following a dog bite to the left iliac fossa. The pain worsened on coughing. No history of coagulopathy, anticoagulant usage, or prior surgeries was present. She had no notable medical history, was not using any long-term drugs, and was a non-smoker.

**Figure 1.**
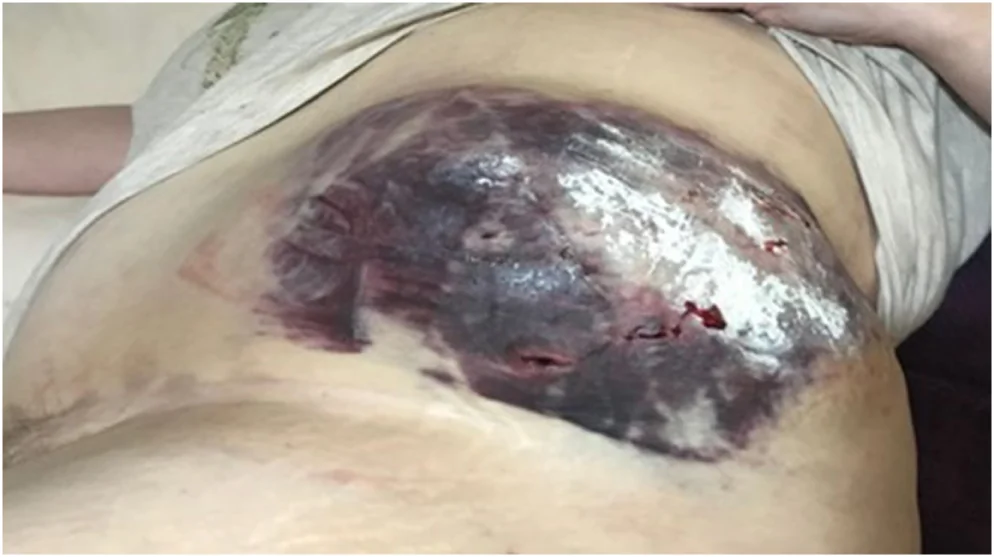
Image taken by the patient on the second day after the dog bite, showing extensive left-sided abdominal bruising with visible dog-bite puncture marks.

The patient reported that while walking her dog, a bulldog attempted to attack her pet. She was bitten in the left iliac fossa while bending to lift her dog. The bulldog was immunized and remained under observation by its owner. There was no specific treatment done at home aside from basic wound cleansing; no formal wound management or dressing was performed by the patient. Upon assessment, her vital signs were steady; she was hemodynamically stable, awake, and afebrile. An examination of the abdomen showed widespread ecchymosis that stretched from the left iliac region to the umbilicus, along with active bleeding from the dog bite site (Fig. 1).

**Figure 2.**
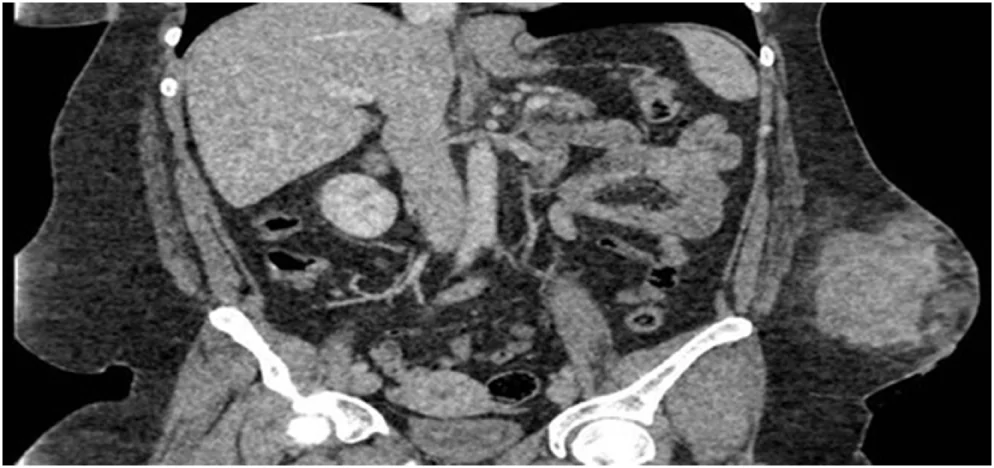
Contrast-enhanced CT scan of the abdomen on admission, showing a 4.7 × 10.8 × 8.9 cm hematoma.

Initial laboratory results showed a C-reactive protein of 93 mg/L and a white blood cell count of 10.4 × 10^9^/L (upper normal limit), suggesting mild-to-severe inflammation that was most likely caused by trauma. A 4.7 × 10.8 × 8.9 cm hematoma in the subcutaneous tissues covering the left oblique muscles with surrounding stranding was visible on an abdominal contrast-enhanced CT scan. There were no foreign bodies, subcutaneous emphysema, or intra-abdominal injuries found (Fig. 2).

**Figure 3.**
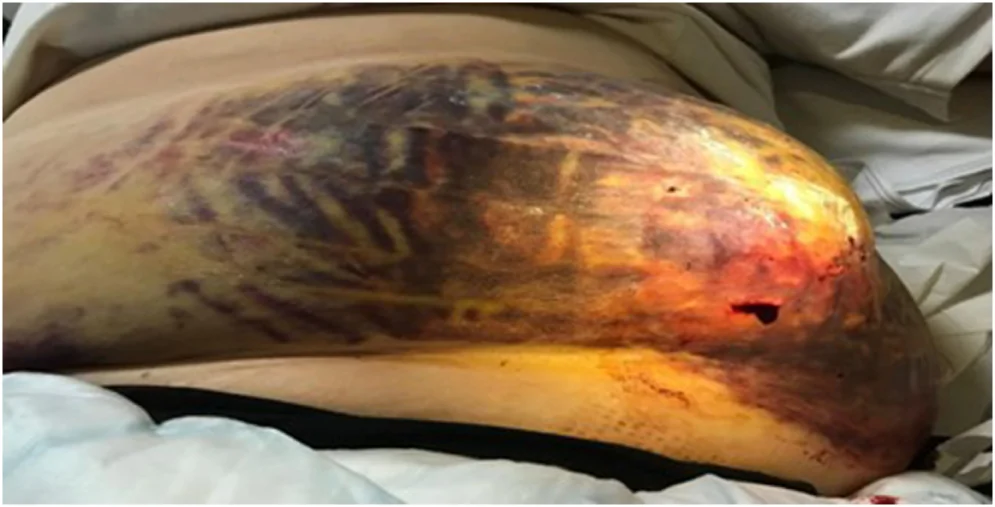
Image taken by the patient on the fourth day after the dog bite, showing progressive enlargement of the hematoma with oozing of dark blood.

Concerns about infection or continuous bleeding were raised as the hematoma enlarged in 4 days post-bite and dark blood began to seep out (Fig. 3). Hemodynamic stability was maintained because bleeding was localized within the subcutaneous plane. Clinically and radiologically, necrotizing soft tissue infection, rectus sheath hematoma, and abdominal wall abscess were excluded.

The general surgery team performed emergency incision and drainage after CT imaging confirmed hematoma’s size and the progression. No visible necrosis was noted on initial examination; necrotic subcutaneous tissue was debrided and about 500 mL of blood was aspirated. The lack of vascular damage or a deeper infection was verified by intraoperative results. No drain was placed as per operative decision, considering the nature of the hematoma and patient’s early discharge.

Wound cultures were not obtained due to the absence of overt infection signs. Empirical antibiotics were administered to cover common dog bite pathogens. Intravenous antibiotics were administered during hospitalization based on our hospital protocol, followed by oral antibiotics upon discharge. She was prescribed amoxicillin and metronidazole for broad-spectrum antibiotic coverage, which targets common dog bite pathogens, and she was given tetanus prophylaxis (REVAXIS and HTIg). Dressing changes were performed daily by community nurses after patient self-discharge. Management was directed by a multidisciplinary team that included advice from infectious diseases, radiology, and surgery. Rabies post-exposure prophylaxis (PEP) was not indicated as the dog was immunized, as per UK guidelines.

Postoperatively, the patient had a stable clinical course, with gradual resolution of tenderness and bruising. She responded effectively to intravenous antibiotics before switching to oral medication, and her vital signs stayed within normal ranges. She was discharged with explicit follow-up instructions for wound monitoring and infection symptoms, and wound care was continued with routine dressing changes.

**Figure 4.**
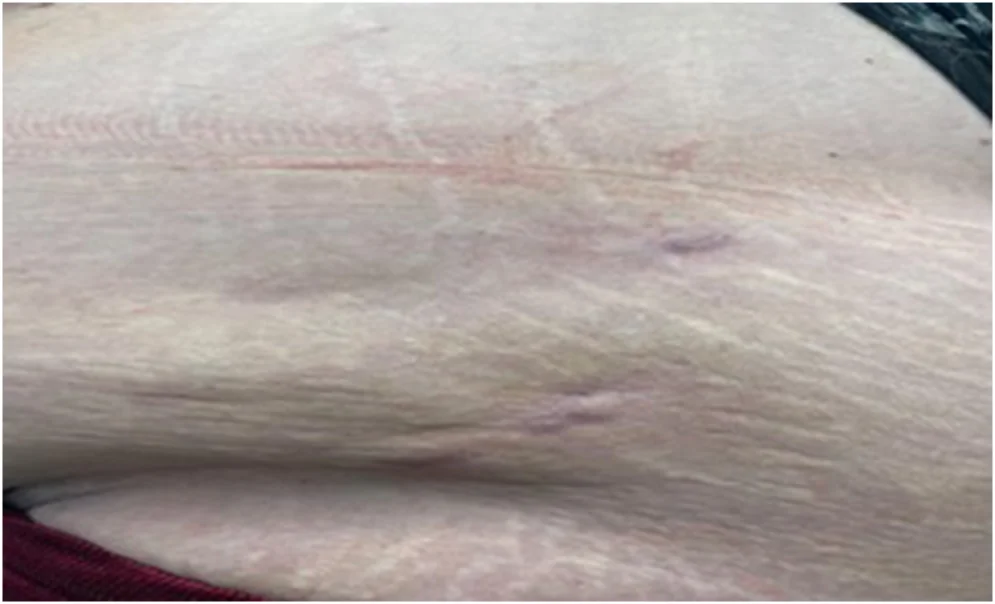
Follow-up image taken 6 months after the dog bite injury, showing complete wound healing.

**Figure 5.**
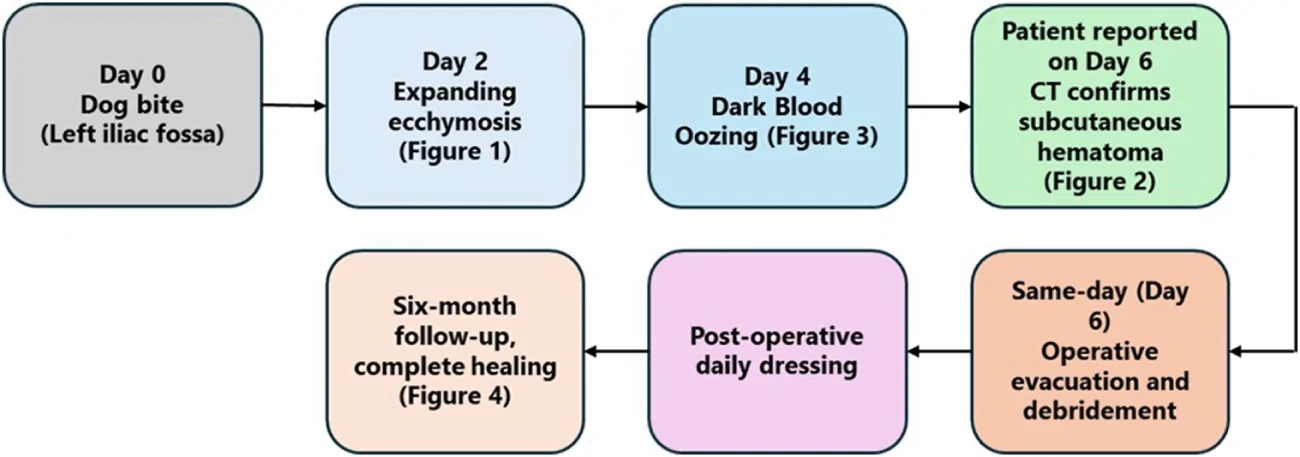
Clinical timeline. The figures are presented in the same sequence in which they were originally provided. Figs. 1 and 3 were provided by the patient. Although the event corresponding to Fig. 2 occurred after Fig. 3 in the clinical timeline, the figures have been labeled according to the order available at the time of submission to maintain consistency with the source material.

The patient reported complete wound healing at a 6-month follow-up, with no hematoma recurrence or infection symptoms (Fig. 4). The clinical timeline on the sequence of events is mentioned in Figure 5.

## Discussion

Dog bites are a significant public health concern worldwide, posing health, economic, and social burdens on communities[12]. While many dog bite injuries are minor, severe bites requiring medical intervention and hospitalization are not uncommon[6].

The severity of dog bite injuries depends on multiple factors, including the location of the bite, the breed of the dog, and the force exerted by the animal’s canines[13]. Although dog bite injuries have decreased in the United States over time, their severity remains a substantial health concern in England[5].

### Mechanism of hematoma formation in this case

**Figure 6.**
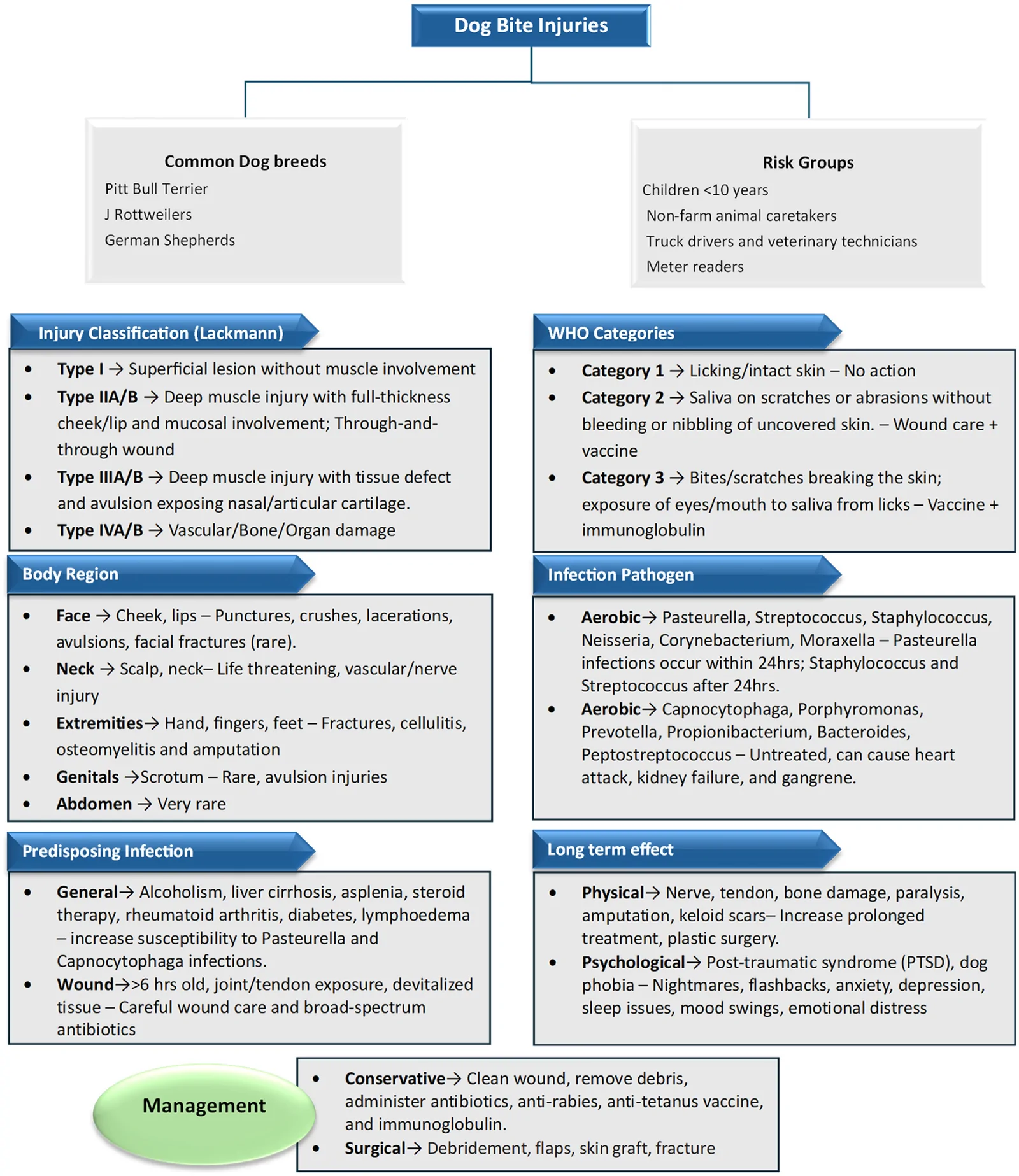
Summary of key points describing dog bite injuries.

This case demonstrates a localized subcutaneous hematoma of the lateral abdominal wall following a dog bite to the left iliac fossa. The blunt penetrating force of canine teeth likely caused shearing of small perforators within the oblique muscle plane, producing progressive oozing rather than brisk arterial hemorrhage. The absence of anticoagulant use or coagulopathy supports trauma-driven vessel injury as the etiology. Clinically, the patient remained hemodynamically stable because bleeding was compartmentalized within the subcutaneous tissues, despite expanding ecchymosis and dark blood oozing observed prior to surgery (Fig. 6).

### Imaging and management decisions

In our case, surgical intervention was performed only after persistent discomfort and hematoma detection on CT scan. A large hematoma with significant bruising and internal bleeding necessitated surgical evacuation to prevent further complications. CT imaging played a vital role in confirming the extent of the injury, allowing for early intervention and anticipatory care. The hematoma was detected 6 days post-bite, enabling timely therapeutic measures before progression to secondary infection or compartment syndrome.

We proceeded to operative evacuation because of progressive enlargement, pain, and ongoing oozing, despite hemodynamic stability. Operative findings confirmed the diagnosis and mitigated the risks associated with continued expansion.

**Operative details**

- **Anesthesia and approach**: General anesthesia; incision and drainage at the most dependent point over the hematoma.
- **Evacuation and debridement**: Approximately 500 mL of dark, partially clotted blood was evacuated. Necrotic subcutaneous tissue overlying the oblique musculature was debrided until healthy tissue margins were reached.
- **Exploration**: No deeper fascial breach, vascular injury, or foreign body was identified; no evidence of necrotizing soft tissue infection.
- **Drain decision**: No drain was placed. This decision was based on (1) cavity collapse after evacuation with good tissue apposition and (2) the patient’s early self-discharge.

Surgical or arterial embolization procedures are generally reserved for cases where conservative management fails, particularly in cases of persistent active bleeding[14]. Although the mortality rate for hematomas requiring surgical intervention is low, it can reach 4% in severe cases[14]. In this patient, minor surgical intervention was sufficient, as no vascular injury or ongoing hemorrhage was observed.

Postoperatively, the patient received daily community dressing, showed stable vitals, and progressed to complete healing at 6-month follow-up.

### Microbiology clarification, cultures, and antibiotic justification

Dog bite wounds are typically polymicrobial, commonly involving *Pasteurella, Streptococcus, Staphylococcus*, and anaerobes such as Bacteroides/Clostridium. In our patient, the lesion represented a closed hematoma without clinical signs of infection (no purulence, no systemic toxicity), and intra-operative findings did not suggest deep infection. Wound cultures were therefore not obtained, consistent with practice in non-infected bite-related soft tissue injuries where empirical prophylaxis is preferred to culture-directed therapy^[^15,16^]^.

Antibiotic selection prioritized broad coverage of common dog bite flora[16]:

- **Amoxicillin**: aerobic gram-positive and gram-negative coverage, including *Pasteurella* (infections developing within 12 hours of dog bite).
- **Metronidazole**: anaerobic coverage (e.g., *Bacteroides* and *Clostridium*; infections appearing more than 24 hours post-bite).

This dual regimen aligns with prophylactic principles for higher-risk presentations (delayed presentation, deep tissue involvement, and hematoma cavity) and the UK context where rabies PEP was not indicated given an immunized domestic dog. Intravenous therapy during hospitalization followed by oral step-down provided adequate coverage while minimizing over-treatment in the absence of infection signs[16].

Two meta-analyses^[^16,17^]^ on antibiotic use in animal bites have yielded mixed results, with variability in infection risk stratification and antibiotic regimens. These studies compared multiple antibiotic regimens, ranging from penicillinase-stable penicillin (e.g., oxacillin) to co-trimoxazole, cefalexin, and phenoxy methyl penicillin, which are not directly applicable to UK clinical practice. In our case, empirical antibiotic therapy with amoxicillin and metronidazole was administered to provide broad-spectrum coverage against common dog bite pathogens, including *Streptococcus, Staphylococcus, Bacteroides*, and *Clostridium* species. This approach aimed to prevent both superficial and deep soft tissue infections.

## Conclusion

Managing dog bite injuries to the lateral abdominal wall requires early recognition of potential oblique muscle damage, hematoma formation, and infection risk, even in the absence of typical risk factors. Urgent CT imaging is crucial for accurate diagnosis, particularly in obese patients, guiding timely surgical intervention when necessary. This case highlights the importance of early hematoma management, ensuring optimal outcomes and preventing complications. Beyond medical care, psychological support for victims and educational awareness for healthcare providers are essential to mitigate long-term impacts and improve clinical management of similar injuries.

## Strengths and limitations

The primary strength of this case report is its novelty, as it describes a unique and rare case of massive abdominal wall hematoma following a dog bite and has important clinical implications for the use of early CT imaging and timely surgical intervention. The delayed presentation and atypical symptoms posed a diagnostic challenge, reinforcing the need for CT imaging in obese patients with acute abdomen where physical examination is unreliable. The case report takes a thorough, multidisciplinary approach to the management of the injury involving radiology, surgery, and infectious disease specialists and follows SCARE guidelines; therefore, it is an educational resource for clinicians treating atypical dog bite injuries. Limitations include that it was a single case report, which means that generalizability is limited; there was no microbiological confirmation via wound cultures. This report adds to the limited evidence on abdominal wall hematomas from dog bites and provides practical insights for clinicians encountering similar rare injuries. Lastly, about the discussion, serial full-body images were not obtained due to explicit privacy constraints agreed in consent.

## Patient perspective

She expressed feeling ignored and scared at the beginning of her treatment due to her learning difficulties. However, by the end of the treatment, she was satisfied with the care she received and hopes that all doctors learn from her experience and take dog bite cases seriously.

## Data Availability

The data that support the findings of this study are available from the corresponding author upon reasonable request.

## References

[1] Taviloglu K Yanar H. Necrotizing fasciitis: strategies for diagnosis and management. World J Emerg Surg 2009;4:19. 10.1186/1749-7922-2-19 PMC1988793 17683625

[2] Cherry WB Mueller PS. Rectus sheath hematoma: review of 126 cases at a single institution. Medicine (Baltimore) 2015;94:e940. 10.1097/01.md.0000216818.13067.5a 16609349

[3] Haagsma JA Graetz N Bolliger I. The global burden of injury: incidence, mortality, disability-adjusted life years and time trends from the Global Burden of Disease study 2013. Inj Prev 2016;22:3–18. 10.1136/injuryprev-2015-041616 PMC4752630 26635210

[4] World Health Organization. Animal bites.

[5] Westgarth C Brooke M Christley RM. How many people have been bitten by dogs? A cross-sectional survey of prevalence, incidence and factors associated with dog bites in a UK community. J Epidemiol Community Health 2018;72:331–36. 10.1136/jech-2017-209330 PMC5868524 29437877

[6] Arluke A Cleary D Patronek G. Defaming rover: error-based latent rhetoric in the medical literature on dog bites. J Appl Anim Welf Sci 2018;21:211–23. 10.1080/10888705.2017.1387550 29068711

[7] Overall KL Love M. Dog bites to humans demography, epidemiology, injury, and risk. J Am Vet Med Assoc 2001;218:1923–34. 10.2460/javma.2001.218.1923 11417736

[8] Tulloch JSP Owczarczak-Garstecka SC Fleming KM. English hospital episode data analysis (1998 2018) reveal that the rise in dog bite hospital admissions is driven by adult cases. Sci Rep 2021;11:1–12. 10.1038/s41598-021-81527-7 PMC7815787 33469116

[9] Morgan M Palmer J. Dog bites. BMJ 2007;334:413–17. 10.1136/bmj.39105.659919.BE PMC1804160 17322257

[10] Daniels DM Ritzi RB O’Neil J Scherer LR. Analysis of nonfatal dog bites in children. J Trauma 2009;66:S17–22. 10.1097/TA.0b013e3181937925 19276721

[11] Maksymowicz K Janeczek A Szotek S. Dog bites in humans in a large urban agglomeration in the southwest of Poland, an analysis of forensic medical records. J Vet Behav 2016;12:20–26.

[12] Karbeyaz K Ayranci U. A Forensic and Medical Evaluation of Dog Bites in a Province of Western Turkey. J Forensic Sci 2014;59:505–09. 10.1111/1556-4029.12343 24313618

[13] Rajshekar M Blizzard L Julian R. The incidence of public sector hospitalisations due to dog bites in Australia 2001-2013. Aust N Z J Public Health 2017;41:377–80. 10.1111/1753-6405.12630 28712151

[14] Loder RT. The demographics of dog bites in the United States. Heliyon 2019;5:e01360. 10.1016/j.heliyon.2019.e01360 PMC6431755 30957043

[15] Cornelissen JMR Hopster H. Dog bites in The Netherlands: A study of victims, injuries, circumstances and aggressors to support evaluation of breed specific legislation. Vet J 2010;186:292–98. 10.1016/j.tvjl.2009.10.001 19879172

[16] Statista.com. Leading pets ranked by estimated population size in the United Kingdom (UK) in 2022.

[17] Evgeniou E Markeson D Iyer S. The management of animal bites in the United Kingdom. Eplasty 2013;13:196–205. PMC3681434 23837110

[18] Fonseca GM Mora E Lucena J. Forensic studies of dog attacks on humans: a focus on bite mark analysis. Res Rep Forensic Med Sci 2015;5:39–51.

[19] Samadov E Ibrahimli A., Mamishov S. Rectus sheath hematoma secondary to domestic violence. Cureus 2021;13:e17058. 10.7759/cureus.17058 PMC8428324 34522536

[20] Rothe K Tsokos M., Handrick W. Animal and Human Bite Wounds. Dtsch Arztebl Int 2015;112:433–42. 10.3238/arztebl.2015.0433 PMC4558873 26179017

